# Mutual Capacitive Sensing Touch Screen Controller for Ultrathin Display with Extended Signal Passband Using Negative Capacitance

**DOI:** 10.3390/s18113637

**Published:** 2018-10-26

**Authors:** Chang-Ju Lee, Jong Kang Park, Canxing Piao, Han-Eol Seo, Jaehyuk Choi, Jung-Hoon Chun

**Affiliations:** 1 College of Information and Communication Engineering, Sungkyunkwan University, Suwon 16419, Korea; changju1.lee@samsung.com (C.-J.L.); jkpark1@skku.edu (J.K.P.); pcanxing@skku.edu (C.P.); 2 Samsung Electronics, Hwaseong 18448, Korea; haneol2.seo@samsung.com

**Keywords:** touch screen panel, flexible display, touch screen controller, negative capacitance, IQ demodulation

## Abstract

Flexible and thin displays for smart devices have a large coupling capacitance between the sensor electrode of the touch screen panel (TSP) and the display electrode. This increased coupling capacitance limits the signal passband to less than 100 kHz, resulting in a significant reduction in the received signal, with a driving frequency of several hundred kilohertz used for noise avoidance. To overcome this problem, we reduced the effective capacitance at the analog front-end by connecting a circuit with a negative capacitance in parallel with the coupling capacitance of the TSP. In addition, the in-phase and quadrature demodulation scheme was used to address the phase fluctuation between the signal and the clock during demodulation. We fabricated a test chip using the 0.35 µm CMOS process and obtained a signal-to-noise ratio of 43.2 dB for a 6 mm diameter metal pillar touch input.

## 1. Introduction

In recent years, ultra-thin and flexible displays have attracted a great deal of attention. The touch screen panel (TSP) on such a thin display must also be very thin, which makes the distance between the touch sensor electrode and the display electrode extremely short, resulting in a large capacitance between the two electrodes. This increased capacitance means the TSP’s signal transfer characteristics can show those of a low pass filter (LPF) with a very low cut-off frequency. Thus, a touch signal with high frequency can be significantly attenuated while passing through the TSP.

We will consider four techniques proposed in previous studies to improve the deterioration of the touch performance by this signal attenuation. Although some of these techniques have not been proposed specifically to address the issue of bandwidth reduction resulting from the increased display coupling capacitance, these potential techniques are considered together as a solution to this problem. The first technique involves sensing the touching of objects using low frequencies where the signal is not attenuated. However, this technique increases the interference with the touch signal from the strong low frequency noises generated by lamps, battery chargers, and home appliances. Of course, using a higher order filter can somewhat compensate for the weakness of the low-frequency driving technique, but if the touch sensing frequency is very close to the noise frequencies, the physical size of the filter would need to be very large. The second method is driving the transmitter (Tx) outputs in touch screen controllers (TSCs) above 10 V to compensate for the signal attenuation [1]. Although this technique is a simple and powerful way to improve the degraded touch performance, it not only requires an additional process for fabricating a high-voltage transistor to drive a high voltage, but also increases the power consumed in the TSP. Furthermore, the high-voltage transistors can considerably increase the size of the Tx block itself, depending on the implementation scheme. The third technique was proposed to speed up the circuit operation by charging the display coupling capacitance using a separate circuit with a large charge-supplying capability rather than the main signal processing path for touch sensing [2]. Because the sensing circuit in the main path only reacts to the amount of signal change by touch, it can be very fast. However, in mutual capacitive sensors, the sensor electrodes for the receiver (Rx) are virtually tied to a fixed voltage. Thus, it is difficult to directly apply this technique to supply a non-zero predetermined charge. The last technique involves improving the sensor electrode of the TSP itself using mesh-shaped metal electrodes instead of conventional face-shaped indium-tin-oxide (ITO) transparent electrodes [3]. The mesh-shape of the sensor electrode can reduce the coupling capacitance between the sensor and display electrodes, and its low resistance can compensate for the effect of the increased capacitance in terms of the bandwidth of the LPF. Nevertheless, in TSCs that use a charge amplifier (CA) scheme because of its excellent immunity to low-frequency noises, the frequency characteristics of the amplifier in the CA stage can limit the bandwidth of the touch sensor [2,4,5,6,7]. This will be discussed in more detail in Section 2.

This paper focuses on how to improve the overall bandwidth of a touch sensor that has been decreased by the increase in the display coupling capacitance of mutual capacitive touch sensors with CA circuits, even though the TSP has a sufficiently large passband using metal-mesh electrodes. As a solution, we propose a scheme to dynamically extend the bandwidth of the signal passband toward higher frequencies by connecting circuits with negative capacitance (NC) to the Rx sensor electrodes to offset the display coupling capacitance.

In addition, we applied an in-phase and quadrature (IQ) demodulation scheme to our design to desensitize the touch sensor to external temperature changes, because numerous metals used as TSP electrodes in ultrathin displays have large temperature coefficient of resistivity (TCR) values [8,9].

The remainder of this paper is organized as follows. In Section 2, we examine the bandwidth reduction phenomenon of the signal due to the increase in the display coupling capacitance of an ultra-thin display, and propose a circuit with NC to overcome this problem. Section 3 describes the implementation of a TSC with an NC circuit in detail. The final section summarizes and discusses the measurement results for a test chip.

## 2. Circuit Analysis of TSP Sensor and Proposed NC Circuit

### 2.1. Passband Lowering Phenomenon by Large Display Coupling Capacitance

Capacitive TSPs have various shapes and structures according to the method used to form the sensor electrode [10]. Among these, on-cell TSPs, where sensor electrodes are formed on the display’s top substrate, and in-cell TSPs, which reuse the display electrodes, are extensively used in ultra-thin displays because they do not substantially increase the thickness of the display. In particular, because on-cell TSPs are driven independently of the display operation, the touch performance cannot be degraded even in a display requiring a high resolution and high frame rate. Therefore, it is very important for the on-cell TSP in an ultra-thin display to maintain the touch performance obtained in thick TSPs.

**Figure 1:**
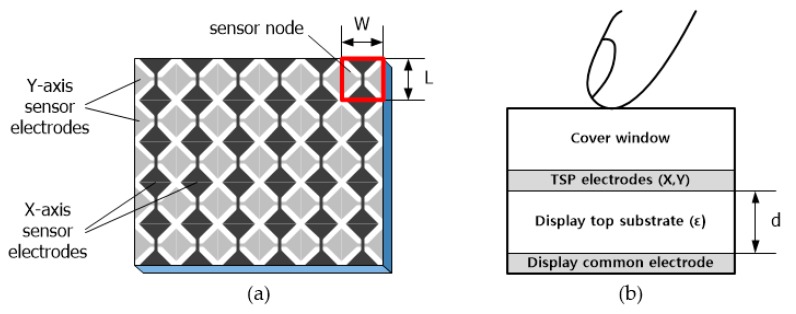
(**a**) Touch screen panel (TSP) with coplanar sensor electrodes and (**b**) vertical structure of display module embedded with TSP.

Figure 1 shows an on-cell TSP with *X*-axis and *Y*-axis sensor electrodes on the same plane, along with the vertical structure of a display module. In the vertical structure, the touch sensor electrodes are usually formed on the opposite side of the display’s common electrode in the top substrate of the display, which corresponds to the encapsulation glass in an OLED display and a color filter substrate in an LCD. At the top of the display, a cover window made of reinforced glass is used to protect the display from scratches or external impact. In conventional thick TSPs, the display’s top substrate and cover glass generally have thicknesses of a few hundred micrometers.

For ultra-thin displays, the thickness of the display’s top substrate, which is denoted by *d*, is the first to be deceased among the stacked layers in Figure 1b. This is because it does not significantly affect the durability of the display module. It has been reported that the thickness has been reduced from tens of micrometers to a few micrometers by replacing the thick glass with a thin plastic film [11]. This decrease in thickness greatly increases the coupling capacitance between the TSP electrodes and the display’s common electrodes, and Table 1 lists the calculated values of the parallel capacitance between two electrodes at a 4 mm × 4 mm TSP sensor node. Here, 4 mm × 4 mm is the size of the most common sensor node for smartphones [12], and the capacitance values are calculated for relative permittivity values of 4 and 10, considering the possibility of using TSP substrates of various materials. From this calculation, we can deduce that a smartphone or tablet with 20–30 channels will have a coupling capacitance ranging from a few hundred picofarads to even a few nanofarads.

Numerous touch sensing integrated circuits (ICs) use a charge amplifier (CA) in the first stage of the analog front-end (AFE) because of its excellent noise immunity [4,5]. They are designed to have the transfer characteristics of a band-pass filter (BPF) with a maximum transfer gain at several hundred kilohertz to block low-frequency noises from power supplies, lamps, and chargers. However, an increase in the display’s coupling capacitance decreases the upper cut-off frequency of the BPF, which moves the passband of the circuit to low frequencies.

There were various technical challenges to overcome this passband lowering problem. One of the most notable achievements was replacing the ITO with a low-resistance metal as a sensor electrode material. Recently, mesh-shaped metal electrodes have become widely used in many TSPs.

**Figure 2:**
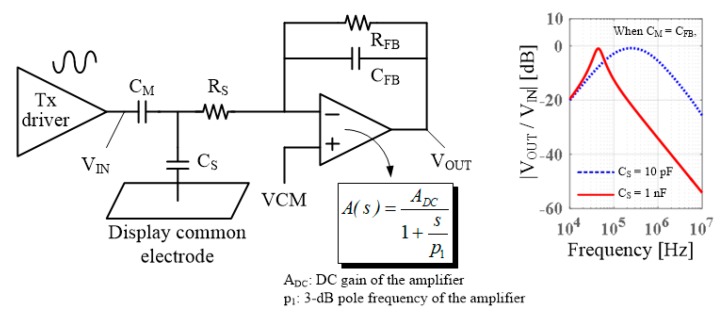
Pass-band lowering phenomenon due to large *C_S_*.

Nevertheless, if the display coupling capacitance is overwhelmingly large, the passband may be decreased because of the CA circuit characteristics of the AFE, even if the bandwidth of the TSP is maintained at the level that existed prior to using low-resistance electrodes. Regardless of the thickness reduction of the TSP, the feedback capacitance inside the CA circuit is not changed much because the mutual capacitance sensed using the feedback capacitance is not significantly affected by the thickness of the TSP. Therefore, the increase in the aforementioned coupling capacitance decreases the feedback gain and finally decreases the bandwidth of the closed-loop circuit, which has an amplifier with one pole in the CA stage. If this bandwidth is smaller than the first pole of the TSP, the upper cut-off frequency of the BPF of the CA stage is pulled downward. Figure 2 shows a typical circuit model of touch sensors which sense mutual capacitance using the CA and the simulation results of the transfer function of a CA stage connected to a TSP for two cases with large and small values for the coupling capacitance between the display and TSP electrodes (*C_S_*). Here, *C_M_* denotes a mutual capacitance between a certain driving electrode and a sensing electrode in a TSP, and *R_S_* denotes a resistance of a sensing electrode. Additionally, in these simulations, we assumed that the amplifier used at the CA stage had a unity gain bandwidth (UGBW) of 10 MHz and that the feedback capacitance of the CA stage (*C_FB_*) was 2 pF, reflecting the design values used in common touch sensors. When *C_S_* is changed from 10 pF to 1 nF, the peak frequency of the passband is significantly lowered. If the passband is lowered by this large *C_S_*, it is necessary to drop the Tx driving frequency, which is approximately several hundred kilohertz with a small *C_S_*, even down to 100 kHz. In this case, the interference of low-frequency noises significantly deteriorates the touch sensing performance.

### 2.2. Design of NC

**Figure 3:**
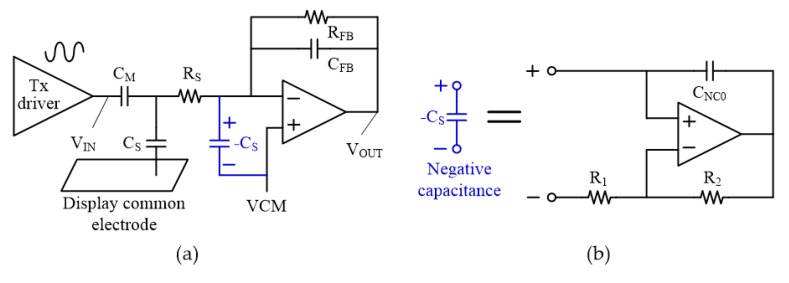
(**a**) Charge amplifier with negative capacitance circuit and (**b**) circuit implementation of negative capacitance (NC).

**Figure 4:**
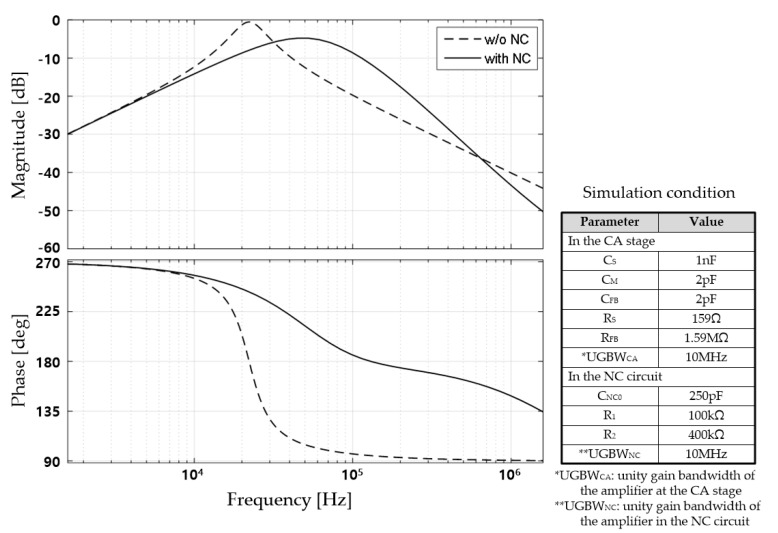
Signal transfer function (STF) at CA stage with and without proposed NC circuit.

To improve the degraded signal transfer characteristics resulting from the large *C_S_*, we can simply increase the bandwidth of the amplifier at the CA stage. However, this method causes a serious increase in power consumption [13]. In our work, we proposed a technique to enlarge the signal passband by arranging an NC generating circuit in parallel with *C_S_*, as described in Figure 3. The NC is generated using a Miller capacitor connected in parallel with the amplifier with a positive gain, as depicted in Figure 3b. Here, the NC value is approximately −*C_NC_*_0_ × (*R*_2_/*R*_1_) where *C_NC_*_0_ is the base feedback capacitance of the proposed NC circuit, and R_1_ and R_2_ correspond to the resistors shown in Figure 3b. As a result, *C_S_* is compensated by the inserted NC, and the passband of the signal transfer function is effectively extended. Figure 4 shows the simulation results for the signal transfer characteristics of the CA stage with and without NC. It demonstrates that the proposed NC circuit can increase the driving frequency of the touch sensor from below 100 kHz to several hundred kilohertz. The increased frequency of the touch driving signal is very effective in suppressing the strong external low-frequency noises.

**Figure 5:**
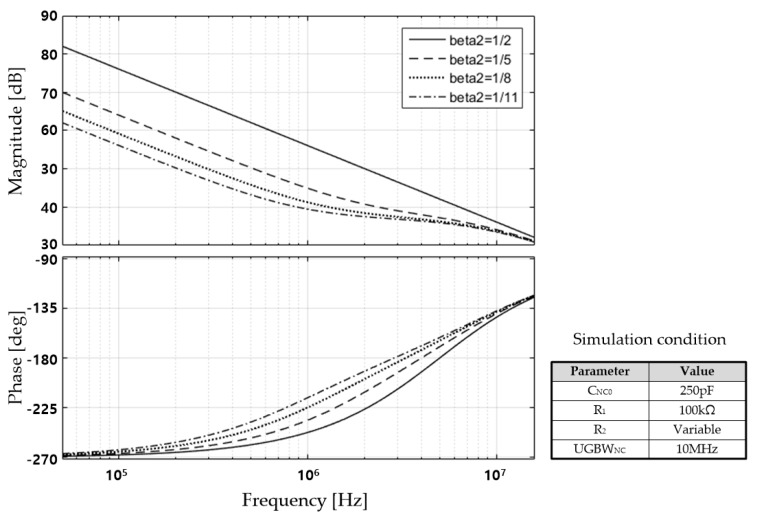
Magnitude and phase characteristics of impedance of proposed NC circuit.

On the other hand, because the proposed *NC* circuit includes an amplifier, the frequency characteristics of the NC are affected by the frequency characteristics of the amplifier. The impedance of the *NC* circuit, including an amplifier with one pole, is derived by Equation (1), and the frequency characteristics are shown in Figure 5.

(1) $$Z_{NC}\left(s\right)=\frac{1}{sC_{NC0}}\cdot \left(\frac{1+A_{NC,amp}\left(s\right)\beta_{2}}{1+A_{NC,amp}\left(s\right)\left(\beta_{2}-1\right)}\right)$$

where $A_{NC,amp}\left(s\right)=\frac{A_{DC2}}{1+\frac{s}{p_{2}}},\;\beta_{2}=\frac{R_{1}}{R_{1}+R_{2}}$, *A_DC_*_2_ and *p*_2_ stand for the DC gain and the 3-dB pole frequency of the amplifier inside the proposed *NC* circuit, respectively. Equation (1) confirms that in the low-frequency region, the *NC* circuit has a negative capacitance of −*C_NC_*_0_ × (*R*_2_/*R*_1_). For reference, this value is equal to the negative value of the product of the Miller gain (*A_M_* = *R*_2_/*R_1_*) and the base feedback capacitance (*C_NC_*_0_). However, the phase of the *NC* circuit moves away from 270° above a certain frequency, as shown in Figure 5.

**Figure 6:**
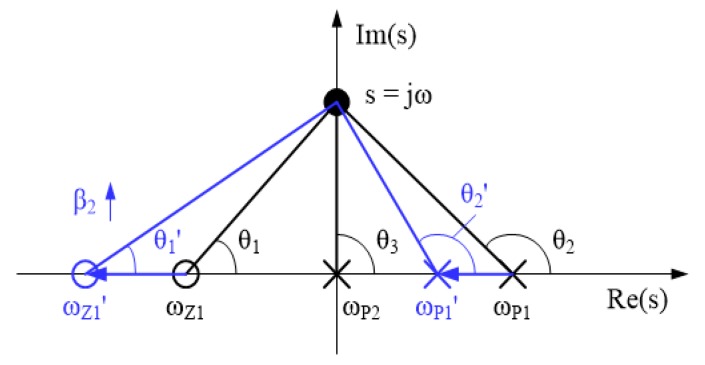
Pole-zero plot of impedance of proposed NC circuit.

To analyze the design factors affecting the characteristics of the NC circuit in more detail, we conducted a pole-zero plot analysis of the impedance of the circuit, as depicted by Figure 6.

In Equation (1), the NC circuit has two poles at the origin and right-half-plane (RHP) and one zero at the left-half-plane (LHP) in the s-domain, and the poles and zero have the values found using Equation (2). In this equation, *ω_u,NC_* stands for the product of the DC gain of the amplifier inside the proposed NC circuit (*A_DC_*_2_) and 3 dB bandwidth (*p*_2_), which means the unity gain bandwidth of the amplifier:

(2) $$\left\{ \begin{array}{l} \omega_{Z1}=-p_{2}-\omega_{u,NC}\cdot \beta_{2},\\ \omega_{P1}=-p_{2}+\omega_{u,NC}\cdot \left(1-\beta_{2}\right),\\ \omega_{P2}=0 \end{array}\right.$$

Then, the phase of the impedance from the pole-zero plot can be obtained by Equation (3).

(3) $$∠Z\left(s\right)=\theta_{1}-\theta_{2}-\theta_{3}=\theta_{1}-\theta_{2}-90{}^{\circ}$$

If the amplifier has a sufficiently large DC gain and the negative feedback gain (*β*_2_) is not very close to 1 or 0, the RHP pole and LHP zero have values near *ω_u,NC_* × (1 − *β*_2_) and −*ω_u,NC_* × *β*_2_, respectively. Meanwhile, *θ*_1_ and *θ*_2_ are almost 0° and 180° at low frequencies, and as the frequency increases, both converge to 90°. This observation explains why the impedance of the proposed circuit with *NC* characteristics at low frequencies gradually changes to a positive capacitance as it goes to high frequencies.

In order to use the high-frequency touch-driving signal in the TSP, the valid region of NC should be sufficiently wide. We can consider two methods to attain the necessary width: using an amplifier with a large UGBW and optimizing the feedback gain setting.

When the UGBW of the amplifier becomes larger, the impedance of the proposed circuit can maintain a negative capacitance characteristic up to a higher frequency region because the RHP pole and LHP zero move away from each other. However, the increase in the UGBW necessarily accompanies an increase in the power consumption of the amplifier. Therefore, it is necessary to design the UGBW of the amplifier to ensure that the NC characteristic is maintained just until the driving frequency of the touch sensor to attain a low-power design.

**Figure 7:**
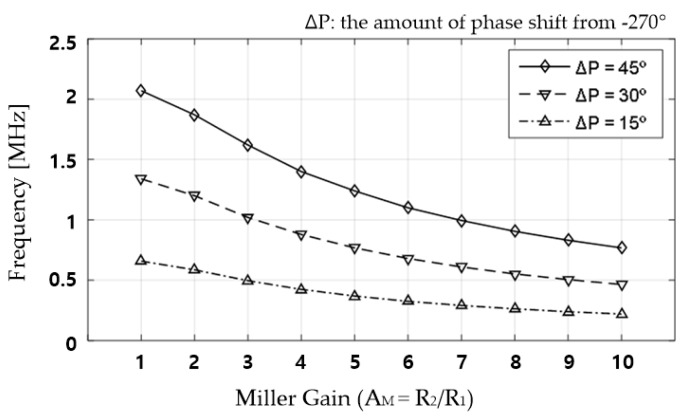
Phase-shifted frequency according to various Miller gains of proposed NC circuit.

As for the selection of the feedback gain, because the RHP pole and LHP zero move in the same direction based on the change in the value of *β*_2_, we simulated a change in the valid region of the NC. When setting the NC value, because the Miller gain rather than the feedback gain provides direct intuition in the circuit analysis and *β*_2_ is equal to 1/(1 − *A_M_*), the Miller gain is used as an independent variable in the result plot. Figure 7 plots the relationship between the frequency with 15°, 30°, and 45° phase shifts from the ideal negative capacitance and the Miller gain of the NC circuit.

In this figure, we can find that the frequency at which the phase shift occurs decreases as the Miller gain increases or the *β*_2_ value of the NC circuit decreases. This is because the closed loop bandwidth of the NC circuit decreases as the Miller gain increases. Therefore, using a small *C_NC_*_0_ and an excessively large Miller gain to obtain a certain NC value is undesirable in terms of the decrease in the valid region of the NC. In contrast, using a large *C_NC_*_0_ and small Miller gain is advantageous for a low-power circuit design, but this combination can increase the chip size. Therefore, it is important to select the appropriate Miller gain considering the product cost.

Consequently, when the proposed circuit is used for an NC, the UGBW of the amplifier, Miller gain of the NC circuit, and *C_NC_*_0_ value should be optimized considering the driving frequency of the touch sensor.

## 3. Full Chip Implementation

### 3.1. Block Diagram of Touch Screen Controller

**Figure 8:**
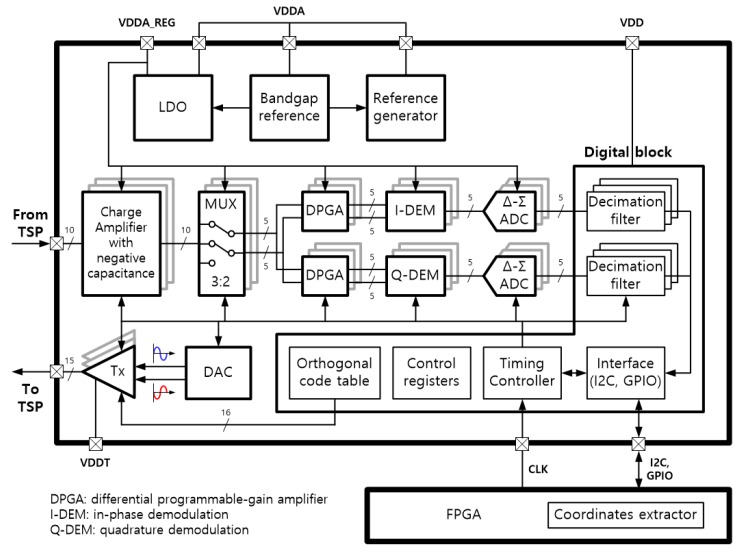
Block diagram of full chip including interfaces with a FPGA chip.

Figure 8 shows a block diagram of a full-chip TSC used for testing. In order to prevent noises generated in other blocks from interfering with the noise-sensitive analog blocks, three separate power sources are supplied to the chip: VDDA for the analog block, VDD for the digital block, and VDDT for the Tx driver in the figure. Furthermore, the voltage regulated by a low drop-out (LDO) circuit is used in the analog circuits located in the touch signal processing path for superior noise performance. The chip communicates with a field programmable gate array (FPGA) chip through inter-integrated circuit (I2C) and general purpose input/output (GPIO) interfaces for the digital processing of touch data to enhance the touching performance and calculation of the coordinates of the touched object.

In the block diagram, the Rx channels of the TSP are connected to the negative inputs of single-ended CAs that include NC circuits. A pair of adjacent CA outputs passing through a 3:2 multiplexer (MUX) is identically input to two differential programmable gain amplifiers (DPGAs) for IQ demodulation. By repeatedly alternating the CA output pairs using the 3:2 MUX, the number of DPGAs can be reduced by nearly half [14]. The differential sensing scheme can not only eliminate the noise common to Rx channels, such as the display and internal bias noise, but also alleviate the newly introduced noise by the NC circuit [6]. The outputs of the upper DPGA in Figure 8 are demodulated with an in-phase clock that has the same triggering edge as the Tx driving pulse, and the outputs of the lower DPGA are demodulated with a clock that is shifted 90° compared to the in-phase clock. Each demodulated output is converted to a digital code by a Δ-Σ analog-to-digital converter (ADC) and then transmitted to the FPGA chip through an I2C communication interface to calculate the touch coordinates.

The digital block is composed of decimation filters for the Δ-Σ ADC, a timing controller, parameter setting registers for the analog IPs, and an orthogonal code table for simultaneous multiple Tx driving operations [15]. The orthogonal code used is a modified Hadamard matrix with the maximum column sum balanced from 4 to 16 Tx lines [16]. The Tx driver consists of a digital-to-analog converter (DAC) and numerous buffers. The DAC generates a sine-wave with almost no harmonics at high frequencies to make the touch data insensitive to environmental changes in the TSP, such as temperature change. Moreover, because the DAC is implemented to simultaneously generate two waves with opposite phases, multiple driving pulses can be simultaneously output according to the orthogonal code by controlling the output switches.

### 3.2. IQ Demodulation

Multiple AFEs with a lock-in architecture for enhancing the signal-to-noise ratio (SNR) are used to demodulate the outputs of the front-end using a clock with a predetermined phase so that the phase of the clock can be locked to the touch signal received for each touch sensor node [4,5]. However, this scheme has the problem that the strength of the demodulated signal is reduced by a temporal phase mismatch between two signals, which can be caused by changes in the device temperature, changes in the parasitic components of the TSP as a result of touch events, and the demodulation clock’s jitter.

In particular, ultra-thin displays such as flexible displays are more sensitive to heat. As previously mentioned, metals with low resistivity are used in ultra-thin displays instead of ITO to ensure a wide bandwidth performance for the TSP. However, most of the metals that can be used in ultra-thin displays have relatively large TCR values of approximately 0.004, which are 20 times greater than that of ITO (0.0002) [8]. This means that thin TSPs using metal electrodes can be more temperature sensitive than previous TSPs. Because the resistance of the sensor electrode determines the cut-off frequency of the passband of the TSP, a large change in the sensor resistance with a change in temperature can have a profound effect on the touch performance.

To evaluate the sensitivity of touch signals to temperature changes in TSPs using metal electrodes, we consider the following example that reflects the characteristics of a typical touch sensor. The TSP forms a second-order LPF with two poles at a cut-off frequency, and the feedback impedance in the CA stage has a pole at the same frequency for low-frequency noise avoidance. If the Tx signal is driven with the cut-off frequency and the temperature of the TSP changes by 25 °C, the phase of the received signal is shifted by 9.04° as a result of the variation in the bandwidth of the passband of the TSP. Then, this phase difference of 9.04° compared to the demodulation clock causes a signal loss of 1.24% in the demodulation process using a square wave. In conventional mutual-capacitive TSPs, because the maximum variation of the mutual capacitance by touch is approximately 10% of the capacitance sensed at no touch, this 1.24% signal reduction after demodulation actually implies a reduction of 12.4% of the meaningful signal for touch sensing, which is a value that can seriously deteriorate the touch performance.

To address the dynamic phase mismatch caused by environmental variations like external temperature changes, conventional demodulation circuits periodically update the phase of the demodulation clock stored in the memory or use a temperature sensor. However, the periodic updating method for the clock phase has an interval in which the touch sensor cannot cope with a sudden temperature change before the next update time. During this interval, the response to a touch may slow down or malfunction. Although the temperature sensor built into the TSC can compensate for the above interval problem, because each sensor node has a different phase delay for the signal based on the temperature change, this method is also limited to precisely compensating for the phase deviation of all the sensor nodes. Furthermore, in the case of displays with self-luminous elements such as OLED displays, a spatial temperature distribution may occur on the TSP depending on the display image, resulting in the failure of the temperature compensation.

**Figure 9:**
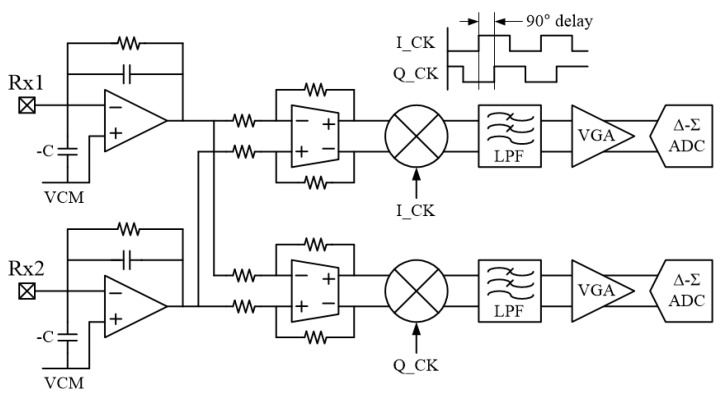
Pair of analog front-end circuits for IQ demodulation.

In this work, we used an IQ demodulation technique to make the touch sensor insensitive to a sudden temperature change. In the IQ demodulation scheme, even if a phase mismatch occurs between two signals, the final value of the touch signal is determined regardless of the phase mismatch using the trigonometric formula of Equation (4).

(4) $${\left(A\;\sin\;\theta \right)}^{2}+{\left(A\;\sin\;\left(\frac{\pi }{2}+\theta \right)\right)}^{2}={\left(A\;\sin\;\theta \right)}^{2}+{\left(A\;\cos\;\theta \right)}^{2}=A^{2}$$

where *θ* is the amount of phase mismatch between the demodulation clock and the signal. In addition, because this technique does not require special chip operation scenarios to lock the phase of the demodulation clock, the demodulation process and circuit implementation can be very simple. Figure 9 illustrates a pair of AFE circuits designed for IQ demodulation, where the squaring and summing operations are performed in the FPGA.

On the other hand, because the IQ demodulation technique requires two signal paths, which are an I-phase signal path and a Q-phase signal path, the demodulation circuits are required twice. However, considering the overhead of the conventional demodulation scheme, which includes the memories of the sensor nodes for storing phase information, multiple clock lines, temperature sensing circuits, complex control logic circuits, and complex chip driving scenarios, the size penalty of the additional blocks for IQ demodulation may not be large.

### 3.3. Remaining Blocks for Analog Signal Processing

The demodulated signal is passed through a 2nd-order LPF to remove the high-frequency components of the demodulated signal and other high-frequency noise components. It is then amplified by a variable gain amplifier (VGA) to minimize the SNR reduction due to the quantization noise in the ADC. Finally, the analog signal is converted to digital code by a 2nd-order incremental Δ-Σ ADC [5,17].

## 4. Measurement Results and Discussion

**Figure 10:**
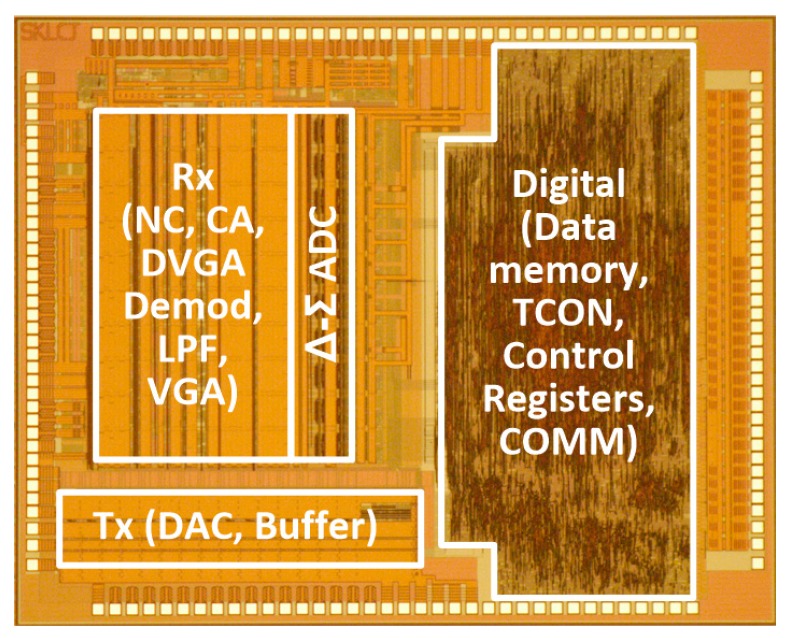
Microphotograph of test chip.

The test chip shown in Figure 10 was implemented using a 0.35 µm 1P4M-CMOS process. It consisted of analog signal processing circuits and Δ-Σ ADCs that could support up to 10 Rx channels, along with various digital circuits, such as those for the data memory, timing controller, control registers, chip-to-chip communication interface circuits, and 15 Tx drivers. The entire Tx and Rx occupy 1.12 mm^2^ and 3.78 mm^2^ respectively. The area of the ADC block is 0.9 mm^2^, and the digital blocks with data memory, timing controller, and registers occupy 5.62 mm^2^.

**Figure 11:**
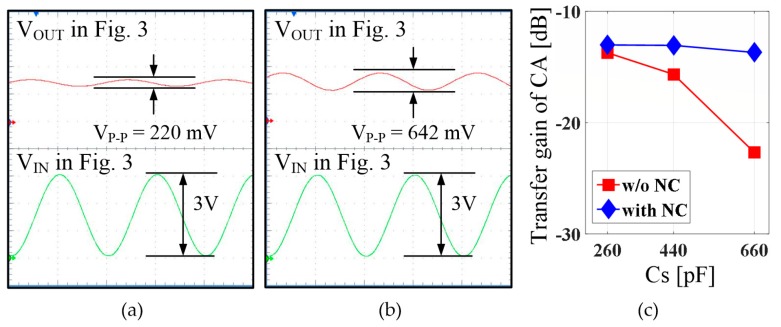
Measured outputs of charge amplifier: (**a**) without NC circuit; (**b**) with NC; and (**c**) gain of CA stage for various *C_S_* values.

We examined the effect of the proposed NC compensation by comparing the outputs with and without the compensation at the CA stage. For comparison, we constructed a test circuit with the same configuration as the equivalent circuit model of the TSP shown in Figure 2. The Tx blocks drove one node of the mutual capacitance with a sine wave that had a peak-to-peak voltage of 3 V and frequency of 200 kHz, and the CA output signals were measured while changing the capacitance corresponding to the display coupling capacitance to various values. As shown in the two measurement results on the left side of Figure 11, when the NC compensation was absent, the CA output had a peak-to-peak voltage of 220 mV, whereas when it was compensated using NC, the output increased approximately three-fold to 642 mV. In addition, Figure 11c shows that the gain of the CA is not substantially reduced even though Cs increases. This result confirmed that the touch performance could be improved using a high-frequency touch signal even in a TSP with a large display coupling capacitance if an NC corresponding to the display coupling capacitance was generated using the proposed NC circuit.

**Figure 12:**
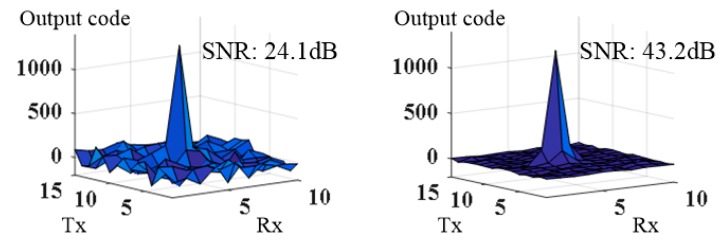
Comparison of touch sensing 2D data without negative capacitance (**left**) and with proposed circuit (**right**).

Next, we compared the ADC outputs to evaluate the SNR of the touch signal on the test chip. In this test, the test chip was connected to a TSP that had 15 Tx and 10 Rx sensor electrodes, and its display coupling capacitance per Rx channel was approximately 660 pF. Figure 12 shows the 2D touch data measured with and without the NC compensation. The VGA outputs were adjusted to be the same in both cases to ensure the same quantization noise for the ADC. We calculated the signal-to-noise ratio (SNR) using Equation (5) [18]:

(5) $$\begin{array}{c} STouch={Signal}_{Touch,AVG100}-{Signal}_{Untouch,AVG100},\\ {NTouch}_{RMS100}=\sqrt{\frac{\sum_{n=0}^{n=99}{\left(Signal\left[n\right]-{Signal}_{Touch}\right)}^{2}}{100}},\\ SNR\left[dB\right]=20\;log\frac{STouch}{{NTouch}_{RMS100}} \end{array}$$

where AVG100 and RMS100 denote the average and the root-mean-square (RMS) of 100 data obtained sequentially, respectively, and *Signal_touch_* is a reference value for measuring the noise RMS value as a longer-term average. In Table 2, the SNR obtained from this study is the average of the SNR values measured at 20 different locations, which are distributed evenly over the TSP. When the NC circuit was used, the SNR was improved by 19.1 dB from 24.1 dB to 43.2 dB. The improvement in the signal transfer characteristics from the C_S_ compensation definitely contributed to this SNR enhancement.

On the other hand, when the NC compensation was applied, the SNR of the 2D touch data was improved by 19 dB, while the touch signal that passed through the CA stage in Figure 11 was increased by 10 dB. This can be explained by the fact that the serious amplification of the device noise and reference noise of the CA-stage amplifier with a large *C_S_* was mitigated. Namely, because the equivalent *C_S_* value was decreased by the NC circuit, the amplification gain of the noise at the CA stage, including the TSP, was reduced, which enhanced the overall SNR of the sensor [5,19]. In addition, because the AFE that used the NC circuit could acquire a sufficiently large signal even with a small signal gain for the CA stage, the gains of the signal amplification stages after the CA stage could be set to small values, and the noise figure of the total chains of the amplifying stages could be improved. As a result, the reduced amplification of the noise in the AFE block also contributed to the enhancement of the overall SNR of the touch sensor.

The main performance of the test chip is summarized in Table 2. It consumed 12.8 mW at a scan rate of 250 Hz and achieved a 43.2 dB SNR on a 6 mm-diameter metal pillar touch. However, there was no information on the display coupling capacitance or thickness of the display top substrate in the state-of-the-art papers, which made it difficult to make a direct comparison with this work. Nevertheless, since the previous experiments were performed on add-on type or thick on-cell type TSPs, the display coupling capacitance is expected to be less than a few tens of picofarads, which may result in better SNR measurement results. Taking this into account, although the device reported here had a slightly lower SNR than those in the two latest studies, the table shows that it had a competitive touch-sensing performance even in a TSP with a large display coupling capacitance of several hundred picofarads.

## Figures and Tables

**Table 1:** Capacitance between sensor electrode and display’s common electrode at sensor node.

*d*	*ε_r_* = 4	*ε_r_* = 10
50 [µm]	5.65 [pF]	14.2 [pF]
10 [µm]	28.3 [pF]	71.0 [pF]

**Table 2:** Comparison of proposed touch sensor with state-of-the-art devices.

	ISSCC 10’ [2]	ISSCC 13’ [4]	JSSC 14’ [5]	TCAS-I 16’ [19]	This Work
Process	90 nm	0.35 µm	0.18 µm	0.18 µm	0.35 µm
TSP Type	Self	Mutual	Mutual	Mutual	Mutual
Channel	^1^ TRX: 24	Tx: 27 Rx: 43	Tx: 12 Rx: 8	Tx: 32 Rx: 10	Tx: 15 Rx: 10
SNR	36 dB	39 dB	60 dB	72 dB	43 dB
Frame Rate	120 Hz	120 Hz	200 Hz	240 Hz	250 Hz
Supply	3 V	3.3 V	2.1–3.3 V	3.3 V	3.3 V
Power	12 mW	18.7 mW	6.26 mW	42.6 mW	12.8 mW
Area	3.65 mm^2^	10.4 mm^2^	2.2 mm^2^	1.25 mm^2^	^2^ 4.89 mm^2^

^1^ TRX = Tx + Rx (Self-capacitive TSP); ^2^ Area of Tx and Rx without the digital block for comparison with other touch sensing ICs.
